# “How do we use the time?” – an observational study measuring the task time distribution of nurses in psychiatric care

**DOI:** 10.1186/s12912-019-0386-3

**Published:** 2019-12-18

**Authors:** Andreas Glantz, Karin Örmon, Boel Sandström

**Affiliations:** 1 Department of General Psychiatry, Psychiatry & Habilitation, Region Skåne, Vuxenpsykiatrimottagning, Elisetorpsvägen 11 B, 232 33 Arlöv, Sweden; 2 Department of Care Science, Faculty of Health and Society, Malmö University, Hälsa och samhälle, Jan Waldenströms gata 25, 214 28 Malmö, Sweden; 3 Department of Health, Faculty of Engineering, Blekinge Institute of Technology (Blekinge Tekniska Högskola), 371 79 Karlskrona, Sweden

**Keywords:** Nursing, Observational study, Psychiatry, Scandinavian and Nordic countries, Time and motion studies

## Abstract

**Background:**

The nurse’s primary task in psychiatric care should be to plan for the patient’s care in cooperation with the patient and spend the time needed to build a relationship. Psychiatric care nurses however claim that they lack the necessary time to communicate with patients. To investigate the validity of such claims, this time-motion study aimed at identifying how nurses working at inpatient psychiatric wards distribute their time between a variety of tasks during a working day.

**Methods:**

During the period of December 2015 and February 2016, a total of 129 h and 23 min of structured observations of 12 nurses were carried out at six inpatient wards at one psychiatric clinic in southern Sweden. Time, frequency of tasks and number of interruptions were recorded and analysed using descriptive statistics.

**Results:**

Administering drugs or medications accounted for the largest part of the measured time (17.5%) followed by indirect care (16%). Relatively little time was spent on direct care, the third largest category in the study (15.3%), while an unexpectedly high proportion of time (11.3%) was spent on ward related tasks. Nurses were also interrupted in 75% of all medication administering tasks.

**Conclusions:**

Nurses working in inpatient psychiatric care spend little time in direct contact with the patients and medication administration is interrupted very often. As a result, it is difficult to establish therapeutic relationships with patients. This is an area of concern for both patient safety and nurses’ job satisfaction.

## Background

Time spent with patients is considered an important factor in defining good psychiatric care [1–3]. Time is also an important factor when nurses and patients are building a trusting relationship [2]. This type of therapeutic relationship is important for the patient’s recovery [4]. The benefits of this kind of relationship between patients and nurses are that they combat self-stigmatization and demoralization [2], predict better short- and long-term patient outcomes [4] and make the patient feel valued [5]. In terms of the therapeutic relationship, patients value good listening and communication [2, 6]. For example, Wyder identified that working partnerships with nurses made patients feel valued. Similarly, Benbenishty and colleagues [7] reported touch, guidance, the investment of time, and nurses promoting healing through their attitudes as vital aspects of the therapeutic relationship. Patients express that continuity of nursing staff as well as interactions leading to trust and a sense of being taken seriously are important for creating a therapeutic relationship [5, 8]. Nurses not having sufficient time, on the other hand, has been described by patients as delaying or impeding their recovery [9]. Patients feel dismissed when nurses are not able to make time for them, including when they are busy with other tasks [2, 5]. In this respect, the lack of time to communicate with patients reported by psychiatric nurses is a concern [3].

The important role of the therapeutic relationship, defined as a partnership with the patient in care, is developed in the Tidal Model [1], a theoretical framework to guide nursing practice. There are ten commitments in the Tidal model, with the ninth emphasizing the requirement of the nurse’s time and attention to establish the relationship. Giving a person the gift of time indicates that the person is important. The question is not “How much time do we have?”, however, but “How do we use the time?”. One assumption permeating the Tidal model is that people are their stories and the nurse’s role is to explore the person’s construction of their own experience through narrative within a therapeutic relationship [1, 10, 11]. Similar assumptions govern the concept of person-centred care which can be described as a partnership between patient and nurses and requires the formation of therapeutic relationships between nurse, patient, and sometimes the patients’ significant others [12, 13]. Person-centred care in a psychiatric setting has in one study been defined as cultural, relational and recovery-oriented [14]. The relational aspect is based on the development of relationships between the nurse and the patient that are therapeutic, supporting, empowering and characterized by closeness and honesty. However, forming that kind of therapeutic relationship requires time, and what many nurses face while trying to adopt a person-centred approach is the difficulty of managing conflicting priorities.

In staffing, the patient-to-nurse ratio is important both for the care of the patients and for nurses’ job satisfaction [15]. In interviews with nurses working in forensic psychiatry, Salzmann-Erikson et al. [16] concluded that devoting time to get to know the patient and moving away from the nurse’s traditional task-driven, productive identity was an important goal in nurse-patient relationships. When examining core needs of patients in relation to nursing care, Gabhann [17] found that patients ask for nurses to spend more time getting to know them and understand what they feel and need. As put forth by Lindqvist et al. [18], there is also a link between the nurses’ involvement in direct care and their intention to stay in or leave their positions, where nurses with more involvement in direct care tend to be more satisfied. The patients’ need and wish for more time together with caring and listening nurses is reported in several studies and resonates with what nurses in psychiatric care consider to be good care [2, 16].

Considering the value of nurses spending time with patients, it is of interest to find out how nurses in psychiatric care spend their working time. Previous time-motion studies investigating the nurse’s task distribution have mainly been carried out in general medical care settings and show that nurses spend 18–40% of their time performing direct patient care tasks [19–22]. However, few studies have been carried out in a psychiatric care context. These studies present varied results but indicate that approximately 30–50% of time measured was spent together with the patient [23, 24]. The review published by Sharac et al. [23] indicates that this figure has been virtually the same over the past 35 years, although the amount of time spent with patients may be misleading as it is reported in that review that only 4–20% of that time is spent on therapeutic interactions. The results from a Swedish study revealed that nurses at a psychiatric clinic spent on average 3 h 15 min per day performing direct care tasks [25]. The study also included data regarding medical, surgical and geriatric wards. No studies focusing exclusively on psychiatric nursing were found. Given the documented value of therapeutic relationships between patients and nurses and the necessity of sufficient time to establish such relationships, the aim of this study was to identify how nurses working at inpatient psychiatric wards in Sweden distribute their time across tasks during a working day.

## Methods

A time motion study design [21] was used utilizing structured observations to measure the time psychiatric nurses spent on a variety of tasks during a working day. This design has been used in similar studies where the purpose has been to describe the time task distribution of hospital staff [19, 21, 22].

The concept of time-motion studies originates from the founder of the scientific management movement, Frederick Taylor [26]. In their most basic form, these studies are performed with the aid of a stopwatch where an observer measures the time it takes for a worker to perform a certain task and which movements are needed. Later time-motion studies have implemented and reported this method in a variety of ways, which causes some difficulty in aggregating the results [27]. In this study, time-motion is defined in its basic form where an observer carries out structured observations and measures the time used, in this case, by a nurse carrying out his or her daily tasks.

Observations are defined as structured when a protocol with a set number of activities to observe is used during a number of predetermined situations or with a number of predetermined participants [28]. Structured observations are useful when data concerning frequency, intensity or duration of activities are collected [28]. The protocol used in this study was guided by the WOMBAT (Work Observation Method By Activity Timing) protocol [21].

### Setting and participants

A total of 12 registered nurses volunteered to participate in the study. Due to time and resource constraints this was essentially a convenience sample [29]. To be included, the nurses had to have at least six months work-life experience from the current context. Three of the nurses were nurse specialists, meaning that they were RNs with an additional one-year advanced level university training aimed at psychiatric care, and typically a master’s degree. Nurses working night shifts were excluded due to the different nature of the tasks carried out at night as opposed to those carried out during the day. None of the nurses who volunteered to participate dropped out. Demographic data is presented in Table 1.

**Table 1:** Demographic data

Demographics	
Age Male Female	26–54 years (median 33 years) *n* = 2 *n* = 10
Years as a registered nurse	9 months-8 years (median 4 years, 9 months)
Years at the current ward	4 months-3 years (median 2 years)
Nurse	*n* = 9
Nurse specialist	*n* = 3

The observations were carried out between December 2015 and February 2016 at a public psychiatric clinic in southern Sweden. The clinic consisted of eight units. Six of the units were selected due to their similarity in offering inpatient care. These six units had a total of 80 beds. Two of the six units specialized in general psychiatry, two specialized in caring for patients suffering from psychosis, one unit treated patients with eating disorders and one treated patients with substance abuse disorders. After receiving permission from the head of clinic, the units were visited by the observers with the purpose of informing both unit managers and nurses about the study. The nurses and managers present received written and oral information concerning the study and written information was left for nurses not present at that time. Written and oral consent was obtained from the nurses willing to participate in the study. Under the Swedish Act concerning the Ethical Review of Research Involving Humans the study did not require ethical approval. The study nevertheless followed the guidelines of the Declaration of Helsinki [30] and the project was approved by the institutional research ethics board at Malmö University (HS 2015, no 4).

### Data collection

Data was collected on number of tasks, the time it took to complete a task and the number of interruptions. Gathering data on the three variables “time”, “frequency” and “interruptions” was aided using an application and a protocol. The nurses were shadowed by one of two observers during the full length of a work shift, which took place either from 7:00 am to 3:00 pm or 1:00 pm to 9:00 pm, both weekdays and weekends. The observations were scheduled so that they would resemble a common working schedule at the clinic. Nine of the nurses were observed once, two nurses were observed twice and one nurse three times. Due to the small sample size, observing three of the nurses more than once was considered necessary.

A protocol based on the WOMBAT work task classification system was used [21]. WOMBAT is a method and software that has proven both valid and reliable [19]. Only the classification system was used, not the WOMBAT software itself. The protocol used in this study aims to classify the nurses’ tasks into eleven categories with definitions for each task category as shown in Table 2.

**Table 2:** Task categories and definitions

Task category	Definitions
Direct care	Tasks directly involving the patient, e.g. direct communication with the patient, nursing procedures, bathing etc.
Indirect care	Tasks indirectly related to the patient, e.g. communication with family, rounds, communication about the patient with other staff, planning care without the patient present etc.
Medication tasks	All tasks related to medication, e.g. preparation, documentation, administration etc.
Documentation	Documentation excluding medication documentation
Professional communication	Communication with other staff members, e.g. patient handover to a different unit, shift handovers etc.
Social	Breaks, meals, personal activities.
Ward related tasks	Ward related tasks not associated with the care of an individual patient e.g. cleaning, staffing, stock resupplying etc.
Training/supervision	Student supervision and training during working hours.
Telephony	All phone calls except personal.
In transit	Non-registered time consisting of time between patients and tasks such as movement from a patient room to the nurse’s office or time spent waiting.
Other	Tasks that did not fit within the above categories.

An application, Ultimate Stopwatch 1.1.5 (Pinetree Software, Portland, US), was used to collect the data. The application, a timing tool developed for sporting events with multiple participants can display up to 90 stopwatches at a time on an Apple iPad. This tool was repurposed to measure time spent on tasks, using eleven simultaneously accessible stopwatches named after the task categories. The software also had the ability to export all the gathered data. Two Apple iPads were used to run the software during observations. Interruptions and the number of tasks carried out were noted on a sheet containing the task categories and examples of most tasks carried out at a psychiatric ward.

Five preparatory pilot observations of a total of 20 h were carried out before the actual study commenced. The purpose of these observations was to identify tasks and evaluate the task classification system, train the observers and make sure the data gathered was consistent between observers. Initially the observations were carried out for a short time and then interrupted in order to compare results. This continued until the gathered data was consistent [19, 21, 22]. Then separate, full work day observations were carried out. The purpose of these longer observations was to evaluate the feasibility of observing for full eight-hour shifts without risking observer fatigue, i.e. the observer getting tired and losing focus. These observations made it clear that observing for eight hours at a time did not cause fatigue and was a feasible approach.

### Statistical analysis

Mean task length and mean time spent per day on each task as well as percentages were calculated using descriptive statistics. The number of tasks, number of interruptions and total measured time was calculated. This analysis is presented by task category. All data was analysed using descriptive statistics in SPSS (IBM SPSS Statistics 23, New York, US) and Excel (Microsoft Excel 15.21, Seattle, US).

## Results

Total time observed amounted to 129 h and 23 min distributed over 16 eight-hour work shifts. A total amount of 968 individual activities were registered. These tasks were part of eleven predetermined task categories. The results for these eleven categories are presented in Table 3. A total of 223 interruptions were recorded.

**Table 3:** Overview of results

Task category	Total time measured	Average, work day^†^	Average, task^‡^	Interruptions, frequency^§^
Medication tasks	22 h 42 m	1 h 24 m	15 m 39 s	75%
Indirect care	20 h 43 m	1 h 17 m	3 m 42 s	16,7%
Direct care	19 h 46 m	1 h 13 m	8 m 21 s	0%
Ward-related tasks	14 h 37 m	0 h 54 m	7 m 15 s	28%
Professional communication	14 h 19 m	0 h 53 m	17 m 33 s	20,4%
Social	12 h 3 m	0 h 45 m	9 m 31 s	6,6%
In transit	8 h 19 m	0 h 31 m	–	–
Documentation	6 h 10 m	0 h 23 m	10 m 0 s	127%
Training/Supervision	5 h 27 m	0 h 20 m	18 m 10 s	28%
Telephony	4 h 34 m	0 h 17 m	2 m 55 s	1%
Other	0 h 48 m	0 h 6 m	5 m 56 s	0%

^**†**^Average total time for an eight-hour work day; ^‡^Average time per task within the category; ^§^How often the nurse on average is interrupted while the task is being performed, in percentage

Observations carried out during mornings (7:00 am to 3:00 pm) accounted for 70% (90 h 33 min) while evening shifts (1:00 pm to 9:00 pm) accounted for the remaining 30% (38 h 50 min). In total, 24% of the observations were conducted during weekends. This distribution reflected a common nursing schedule at the clinic.

Nurses spent on average 17.5% of their work day performing medication tasks and all in all 22 h and 42 min was registered in this task category. This makes medication tasks the largest of the observed categories. A number of 87 medication tasks were carried out and an average of 15 min and 39 s was spent on each occasion. A total of 65 interruptions were registered which means that an interruption occurred on approximately 75% of all medication occasions.

The analysis showed that the indirect care task category was the second largest with 16% of the nurses’ work day spent on tasks such as doing rounds or talking about the patients’ care with other staff or with relatives. In all, 20 h and 43 min was registered in this category and the tasks lasted on average 3 min and 42 s. Time spent on tasks categorized as direct care made up for the third largest category. The observations showed that 15.3% of the work day was spent on tasks with the patient directly involved, such as spending time with the patient, planning care in collaboration with the patient or drawing blood. All in all, 142 tasks with an average of 8 min 21 s per task were registered.

Wardrelated tasks, including cleaning, staffing activities or reading e-mails accounted for 11.3% of total time registered. Nurses spent on average 54 min per working day on activities in this category. Activities related to documentation accounted for 4.8% of all registered time but there were 47 registered interruptions and only 37 registered tasks which means this task category had the highest proportion of interruptions.

## Discussion

The purpose of this observational study was to examine how nurses working at inpatient psychiatric wards distribute their time across different activities during a working day. The 129 h of data registered showed that nurses at the clinic under study spent most of their time performing medication-related tasks. Talking about and planning the care of the patients was the second largest category and direct care came in as the third largest category.

A noteworthy result was that time spent on direct care (15.3%) was much lower in this study compared to other studies where figures ranging from 18 to 40.3% have been reported [20–22, 24, 31–33]. In a previous study, Furåker [25] showed that nurses at a psychiatric clinic in Sweden spent on average 3 h 15 min per day performing different direct care tasks. The results in the present study show a considerably lower figure at an average of 1 h 13 min per working day. The average direct care task lasted only about eight minutes which casts into doubt whether it is possible to create trusting and therapeutic relationships with the patients in such a short time. Qualitative research shows that in general, patients desire more time spent together with nurses [34] and invested in relationship building [17]. Instead, at a time when person-centred care is emphasized, this study indicates that the care provided by psychiatric nurses is moving in the opposite direction.

The observed nurses spent slightly more time performing indirect care tasks than tasks where the patient was present. These indirect care activities often consisted of planning the care of the patient without the patient present. The direct care category however did not include many planning tasks. It is therefore possible to draw the conclusion that most of the planning of the patient’s care was carried out without the patient’s direct involvement. This is in violation of the ideal of person-centred care [13] and also contravenes with what patients consider to be of importance in good quality care [2, 5, 8]. A review of papers using person-centred care in relation to in-patient psychiatry showed the significance of patient involvement, a therapeutic milieu and relationships that are characterised by closeness, engagement and trust [14]. In order to promote a more person-centred approach to psychiatric caring, patients should be given the opportunity to be more involved in planning their care. However, this calls into question whether the work conditions of psychiatric nurses in Sweden really allows a development towards person-centred care or if in reality administrative tasks are given precedence.

Not unexpected, medication tasks constituted the largest of the observed categories and nurses spent on average 17.5% of their work day performing medication tasks. Interruptions during medication tasks are common [22, 35, 36] and this study was no exception. Interruptions during medication tasks is believed to be one of the main factors associated with medicine administration errors [37, 38] which should be a reason for management to reduce the occurrence of interruptions.

The result from this study show a tendency within the organization of focusing more on *doing* than *being*. An orientation towards doing is more focused on tasks and working hard towards a result than taking time to reflect and focus on the here and now [39]. This is evident in the relatively high percentages of time spent on ward-related activities in comparison with the very low percentage spent on training. Without the time to reflect and access and read research, professional development and implementation of new techniques and procedures will prove difficult The distribution of time spent on ward-related tasks, in this study reported at 11.3%, was higher than in previous studies with figures ranging from 4 to 8% [20–22, 31, 33]. There may be a reason for nurses to ask themselves the question “How do we use the time?”

The nurses included in this study had relatively short experience working at their respective wards, with 3 years being the longest any nurse had been working at the same ward. Benner’s historically important work on nurses’ levels of expertise [40] highlights the correlation between the nurse’s experience and her skill level as well as what method of work characterises these different levels. Comparing the experience and skill levels put forth in Benner’s work with the demographic information in this study, the average level of experience could be classified as “Advanced beginner” (0–2 years of experience working at the present ward). Advanced beginners lack confidence in prioritizing and seeing the “big picture” and tend to focus on tasks that are characterised by strict guidelines and clear routines. In this study, several of those tasks can be found in the “Medication” and “Ward-related” categories. Although being able to follow guidelines or protocols does not equate expertise, experience alone is not equal to expertise either. Christensen and Hewitt-Taylor [41] argue that expertise is an amalgamation of knowledge and skills, intuition and experience. There is also evidence that the context in which nurses act affect clinical nursing expertise [42]. The higher the proportion of nurses with academic training the better the odds of nurses reporting a more advanced level of expertise [42]. Against this background, the observed nurses’ level of expertise could provide a possible explanation for why these categories were more pronounced in this study compared to other studies. This also highlights the importance of the manner in which beginner nurses are introduced to their profession by more experienced nurses.

The imbalance between direct care and other tasks might be a reflection of various trends in mental health care, including for example denuding of staffing levels. Research shows that inadequate staffing levels are seen as antithetical to the therapeutic alliance and might lead to an over-reliance on pharmacology [44].

This study was designed as a time-motion study with structured observations. This method gives valuable insight into how nurses distribute their time. Studies comparing observations with self-reporting suggest that there are greater margins of error with self-reporting and that staff more readily accepted observations compared to self-reporting [31, 43]. An advantage in this study was that the observers themselves were registered nurses with psychiatric care experience and hence familiar with the tasks carried out, making it easier to quickly identify the observed tasks and thus increase reliability.

Observing a whole work shift has several advantages over work sampling. One advantage is that the risk of missing certain tasks that the nurse may be postponing to the end of the shift, such as documenting, is eliminated entirely. Other tasks are carried out at certain hours of the day and are also at risk of being excluded if work sampling is utilized. Another advantage of longer observations is that the participant may habituate to the presence of the observer which reduces the risk of a Hawthorne effect, that is, the risk that participants change their behaviour due to knowing that they are being studied. There is an increased risk of observer fatigue and several time-motion studies have limited the observations to 60 or 90 min at a time, but there is little evidence to support the notion that 90 min is an upper limit [19]. Nevertheless, this risk cannot be entirely disregarded.

There were several occasions where the nurse performed multiple tasks at once. In this case, the observers only registered the first task. A second simultaneous task was only registered if the first task was interrupted. This approach has been used in previous studies [24]. Using the application on a tablet computer was advantageous compared to a regular stop-watch and note-taking procedure. It is considerably faster as there is no need to note the observed time and there is also less risk of the observer mistyping. A disadvantage was in regard to phone calls. Since the observer could not know in advance whether the nurse talked to for example a patient, a relative or another caregiver, it was impossible to track this information with the application and a separate category for phone calls was implemented. With a traditional stop-watch this would not have been an issue. Despite this, the advantages of the application outweighed the possible advantages of using a stop-watch.

### Limitations

The foremost limitation is that the observations took place at wards with different specialisations. Two of the wards focused on care for patients suffering from psychosis-related disorders, one ward focused on substance abuse disorders, one on eating disorders and two on general psychiatry. There were variations regarding how tasks were carried out at the different wards and how time spent on these tasks was distributed. Since the results of this study are presented as averages, it is possible to generalize the results to some extent, despite variation of care context.

Another limitation is the fact that two tasks carried out simultaneously could not be recorded. It is probable that for instance some medication tasks also presented opportunities for the nurses to interact with the patients and would have constituted as direct care tasks as well.

Although direct, continuous observations are a useful approach to gathering data, in this case the observation scenarios were complex and inter-observer reliability is a concern [27]. To help deal with this, pilot observations were conducted. In addition, the fact that the observers had relevant clinical backgrounds should help improve reliability [45].

There are also limitations to this study concerning participant reactivity. Direct observation may incur what is sometimes dubbed the Hawthorne effect, where participants change their behaviour due to knowing that they are being studied. To offset the occurrence of a Hawthorne effect, all participants were informed that the observations were not centred on their quality of work and that the study was not initiated by their employer. Although it is difficult to rule out the Hawthorne effect completely, recent studies seem to indicate that it may not be as prevalent as previously believed [31, 46].

## Conclusions

This study has identified several potential areas of concern in Swedish psychiatric nursing. The amount of time spent on direct nursing tasks was almost as low as a third of that expected compared to previous time-motion studies carried out in psychiatric settings, whereas a relatively large amount of time was spent on ward related tasks. This could be a result of the short work experiences of the nurses in this study but also the lack of nurse specialists. Another area of concern are the numerous interruptions nurses experienced during medication tasks.

Future studies should focus on detailing the content and quality of psychiatric nursing tasks as well as the patients’ experiences of the care delivered, to establish how time spent one-on-one with patients is used and to what benefit to the patients. Further time-motion studies focused on specific specialities might also be of importance in order to verify these results and make them more generalizable.

### Relevance for clinical practice

This examination of nurses’ task distribution points to a need for change in the psychiatric inpatient care in Sweden. The time nurses spend with patients needs to be increased in order to create and maintain therapeutic relationships that promote trust and well-being. By spending more time with the patients, the nurses will increase their knowledge of the patients’ lives, which is in line with Barker’s Tidal model. An additional benefit is the likelihood that decreasing the time spent on ward related tasks in favour of direct care may increase job satisfaction among nurses and decrease staff turnover. Further, finding ways to increase the nurses’ level of expertise should be of benefit for nurses and patients alike. Finally, the number of interruptions of medication tasks is a major patient safety concern that needs to be addressed.

## Abbreviations

WHO: World Health Organization
WOMBAT: Work Observation Method by Activity Timing
